# Tibial insert design significantly alters knee kinematics using a single cruciate-retaining total knee implant: an in vitro study

**DOI:** 10.1302/2633-1462.57.BJO-2024-0033.R1

**Published:** 2024-07-18

**Authors:** Martin Faschingbauer, Jakob Hambrecht, Jonas Schwer, John R. Martin, Heiko Reichel, Andreas Seitz

**Affiliations:** 1 Department of Orthopedic Surgery, RKU, University of Ulm, Ulm, Germany; 2 Institute of Orthopaedic Research and Biomechanics, University of Ulm, Ulm, Germany; 3 Vanderbilt University Medical Center, Nashville, Tennessee, USA

**Keywords:** Total knee arthroplasty (TKA), Knee kinematics, Insert, Biomechanics, knee kinematics, Patella, tibia, femur, coronal alignment, primary total knee arthroplasty, knee implant, cadaveric knee, simulator, flexion

## Abstract

**Aims:**

Patient dissatisfaction is not uncommon following primary total knee arthroplasty. One proposed method to alleviate this is by improving knee kinematics. Therefore, we aimed to answer the following research question: are there significant differences in knee kinematics based on the design of the tibial insert (cruciate-retaining (CR), ultra-congruent (UC), or medial congruent (MC))?

**Methods:**

Overall, 15 cadaveric knee joints were examined with a CR implant with three different tibial inserts (CR, UC, and MC) using an established knee joint simulator. The effects on coronal alignment, medial and lateral femoral roll back, femorotibial rotation, bony rotations (femur, tibia, and patella), and patellofemoral length ratios were determined.

**Results:**

No statistically significant differences were found regarding coronal alignment (p = 0.087 to p = 0.832). The medial congruent insert demonstrated restricted femoral roll back (mean medial 37.57 mm; lateral 36.34 mm), while the CR insert demonstrated the greatest roll back (medial 42.21 mm; lateral 37.88 mm; p < 0.001, respectively). Femorotibial rotation was greatest with the CR insert with 2.45° (SD 4.75°), then the UC insert with 1.31° (SD 4.15°; p < 0.001), and lowest with the medial congruent insert with 0.8° (SD 4.24°; p < 0.001). The most pronounced patella shift, but lowest patellar rotation, was noted with the CR insert.

**Conclusion:**

The MC insert demonstrated the highest level of constraint of these inserts. Femoral roll back, femorotibial rotation, and single bony rotations were lowest with the MC insert. The patella showed less shifting with the MC insert, but there was significantly increased rotation. While the medial congruent insert was found to have highest constraint, it remains uncertain if this implant recreates native knee kinematics or if this will result in improved patient satisfaction.

Cite this article: *Bone Jt Open* 2024;5(7):592–600.

## Introduction

While total knee arthroplasty (TKA) is associated with excellent results, a subset of patients remain dissatisfied.^1^ Even with improvements in surgical technique and materials, managing patients’ expectations appears to be the key to successful outcomes.^2,3^ In recent years, different alignment variations (mechanical alignment, restricted kinematic alignment) have been used in an attempt to improve outcomes.^4^ In addition to alignment changes, a variety of implant changes have been developed to try and improve clinical outcomes. For example, sales of medial pivot implants and dual pivot implants have increased considerably in the USA (such as Empowr, DJO, USA; Evolution, MicroPort, China; and GMK Sphere, Medacta, USA). Recently, some manufacturers have developed a supplemental tibial insert to their existing implants in an attempt to recreate more “normal” kinematics (e.g. Persona; Zimmer Biomet, USA).

Due to the persistence of dissatisfaction following primary TKA, a variety of technique and implant changes are being explored. One common approach is to try to reproduce natural kinematics. This can be accomplished via various alignment options.^4,5^ Alternatively, this can occur through changes to the component design, including modifications of the tibial insert geometry. In particular, the notable increase in the number of cases with pivoting TKAs should be mentioned.^6^ Attempts are being made to generate in vivo data to demonstrate the kinematic changes with different insert designs.^7,8^ However, the “normal” kinematics of the knee with various inserts are largely unknown. Furthermore, the 2D to 3D registration analysis shows some variability, which may be a reason for a lack of prior kinematic studies.^9^ So far, no kinematics can be read here preoperatively either.

The cruciate-retaining (CR) and posterior-stabilized (PS) designs have been considered the workhorses of primary TKA implants. However, with the CR implants, a few variations have been introduced. The ultra-congruent (UC) insert has been increasingly used by surgeons who prefer a gap-balancing technique. Some surgeons use the UC insert as their standard insert and routinely resect the posterior cruciate ligament (PCL) in a cruciate-sacrificing (CS) procedure.^10^ The rationale for this surgical technique is that the stability is obtained via the conformity of the UC insert. More recently, a polyethylene insert was designed with a more constrained medial and less constrained lateral geometry (medial congruent (MC) insert). The design feature attempts to keep the medial compartment more stable and to allow the lateral compartment more movement. These design features are intended to better replicate the natural knee kinematics with the intent that they will result in improved outcomes after TKA.

Currently, there is a paucity of data exploring the kinematic differences among these three tibial inserts within a single implant design, the CR TKA. Thus, reliance must be placed on the manufacturer’s specifications of the implant’s geometry. For this reason, we designed the following study to determine how changing the tibial insert for a single manufacturer’s CR TKA changes the following kinematic parameters: 1) coronal alignment; 2) medial and lateral femoral roll back; 3) femorotibial rotation; 4) bony rotations (femur, tibia, and patella); and 5) patellofemoral motion.

This study focuses on various CR inserts. Although there are also interesting research questions in the PS setting, such as PS standard insert versus PS high flexion insert or condylar constraint insert versus PS insert, they cannot be addressed here and may be reserved for another research project.

## Methods

This study protocol was approved by the ethics committee of the University of Ulm, Germany. The requirement for informed consent was waived as this was a cadaveric study.

### Biomechanical experiment

The full biomechanical set-up was previously presented in a supplementary document and published by Faschingbauer et al.^11,12^ A short summary is described here for clarity: 15 fresh-frozen whole leg specimens (the lower limb, from the femoral head to the forefoot) were used for in vitro testing. The inclusion criteria for the study were osteoarthritis Kellgren and Lawrence grade 2 or higher,^13^ no history of surgery on the knee joint, no fractures, and no arthroplasty of the knee or hip joint. Further demographic data of the donors were provided by Science Care, USA (Supplementary Table i).

**Fig. 1:**
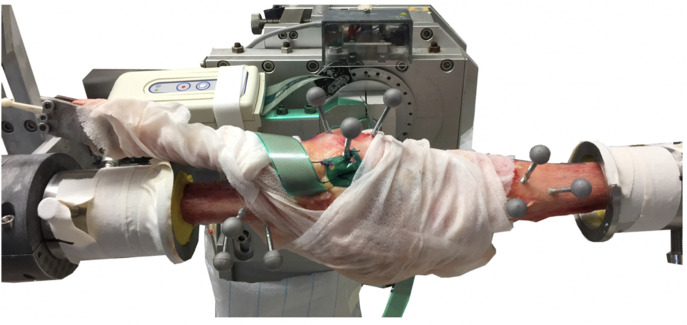
Clamped specimen at the starting position (full extension (shown on the right side the tibia, and the top patella on the left femur)) in the knee simulator. The markers are clearly visible for the OptiTrack system (NaturalPoint, USA), the Tekscan films (Tekscan, USA) are derived via an extra PC, and the preload on the quadriceps tendon is applied.

The experimental setup was guided by the following sequence as first described by Victor et al.^14^ After thawing of the specimen for 24 hours, dissection of the lower limbs was performed, and the reference markers were mounted on the femur, tibia, and patella. Next, a CT of the lower limbs including reference markers as well as a radiograph to determine the tibial axis were performed. Subsequently, the knee was harvested by resecting the proximal femur and distal tibia. Utilizing a mechanical alignment technique, a CR TKA was cemented in place (Persona; Zimmer Biomet). After the femoral and tibial components were cemented in place, a computer program randomly assigned the order of tibial insert selection (CR, UC, MC). The harvested specimen, including the implanted TKA and reference markers, was placed within the knee simulator (Figure 1). Simultaneously, while the knee simulator was ranging the knee, the anatomical landmarks were recorded using OptiTrack (NaturalPoint, USA). The knee kinematics were subsequently measured with each insert sequentially in the order that was randomly assigned.

**Fig. 2:**
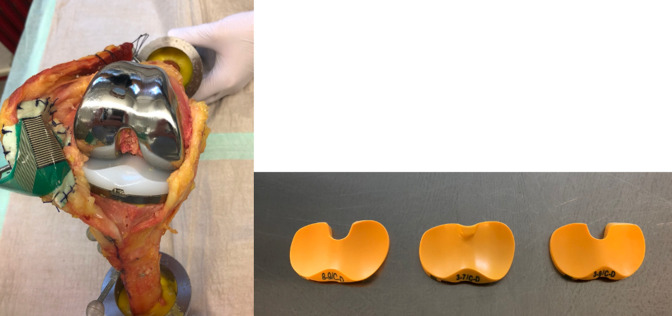
The left photograph shows the standard cruciate-retaining (CR) total knee arthroplasty implanted after mechanical alignment. The inserts used are shown in the photograph on the right: the medial congruent insert on the left with a deepened medial plateau and a flattened lateral plateau, the ultra-congruent insert in the centre with medial and lateral conformity, and the standard CR insert on the right.

The three types of insert used are displayed in Figure 2.

### Measurement of knee kinematics

**Fig. 3:**
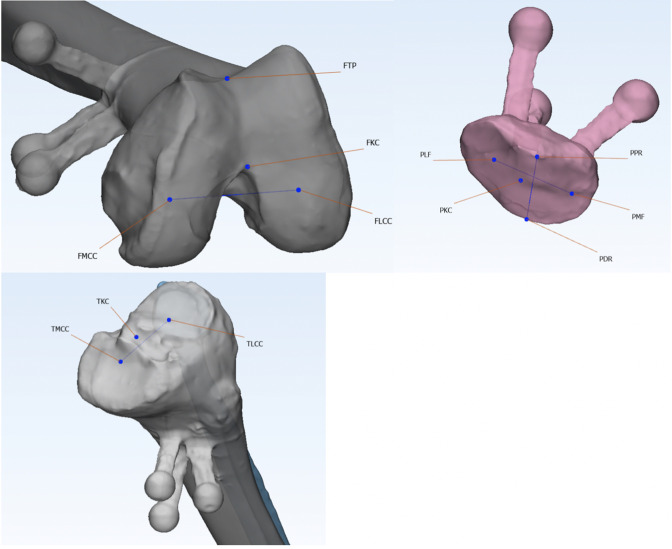
Femoral, tibial, and patellar landmarks: In a clockwise direction, the 3D reconstructions of the femur, patella, and tibia are shown. FKC, centre of the intertrochlear notch; FLCC, centre of the lateral femoral condyle; FMCC, centre of the medial femoral condyle; FTP, deepest point of the trochlea groove; PDR, most distal point of patella's ridge; PKC, centre of the patella; PPR, most proximal point of patella's ridge; TLCC, centre of the lateral tibia plateau; TMCC, centre of the medial tibia plateau.

Digital Imaging and Communications in Medicine (DICOM) data were generated using conventional CT and processed with a 3D visualization software (Mimics Innovation Suite 23; Materialize, Belgium) to visualize the 3D surfaces of the femur, tibia, and patella (Figure 3). Before performing the biomechanical tests, the landmarks were attached to the 3D model (Supplementary Figure a), as described by Victor et al.^14^ During testing, the positions of the landmarks on the 3D model (average of approximately 4,500 tracepoints at 120 recorded frames per second) were calculated (Mathcad; Parametric Technology, USA; and MatLab; MathWorks, USA) and divided by 10° intervals each. Supplementary Figure a shows the relationships of the various femoral, tibial, and patellar axes to the generated planes, using the landmarks described by Victor et al.^14^ The third flexion extension cycle at the knee simulator was used for the statistical analyses. The data analysis includes values ranging from 0° extension to 120° flexion.

The following parameters were then recorded as published in previous studies: coronal axis (°, knee simulator), medial and lateral femoral roll back (mm, marker tracking), femorotibial rotation (°, knee simulator), single femur, tibia, and patella rotation (°, marker tracking), patella distances (mm, marker tracking), and the patella shift (mm, marker tracking).^11,12,14^

### Statistical analysis

All 15 knees were tested with all three different inserts. Data were checked for normal Gaussian distribution (SPSS v. 24; IBM, USA). If normal distribution of the data was found, the data were assessed using a one-way analysis of variance. The homogeneity of the variance was tested by Levene’s test. If homogeneity could not be shown, the Brown-Forsythe test was used. A p-value < 0.05 was considered statistically significant. The mean values of all variables using a CR insert, a MC insert, and a UC insert were compared (CR vs MC; CR vs UC; and MC vs UC).

## Results

### Coronal alignment

When all 15 knee joints were combined and sorted by degrees of flexion (each in 10° intervals), there were no significant differences in coronal alignment between the individual inserts (Table I). Looking at each knee joint individually, there were statistically significant differences between CR and MC inserts in 10/15 knees, between CR and UC inserts in 10/15 knees, and between MC and UC inserts in 6/15 knees (Supplementary Table ii). Mean ranges averaged 5.13° (-2.74° to 8.21°) for MC, 5.22° (-1.3° to 8.8°) for CR, and 5.27° (-3.42° to 8.75°) for UC, and showed no statistical significance.

**Table I.** Varus/valgus alignment divided into degrees of flexion with a 10° interval, showing no statistically significant differences. On the contrary, femorotibial rotations consistently shows significances.

Flexion, °	Mean varus/valgus, ° (SD)			p-value*	Femorotibial rotation, ° (SD)			p-value*
	**CR**	**MC**	**UC**		**CR**	**MC**	**UC**	
0 to 10	1.97 (1.23)	2.02 (1.35)	2.13 (1.29)	0.087	0.33 (0.8)	-0.42 (1.5)	-0.31 (1.31)	< 0.001
10 to 20	2.14 (1.51)	2.2 (1.63)	2.26 (1.57)	0.166	1.41 (2.06)	-0.05 (2.06)	0.16 (2.0)	< 0.001
20 to 30	2.06 (1.83)	2.12 (1.96)	2.1 (1.95)	0.652	2.14 (3.06)	0.75 (2.8)	1.03 (2.73)	< 0.001
30 to 40	1.85 (2.1)	1.91 (2.27)	1.88 (2.28)	0.832	2.48 (3.59)	1.07 (2.98)	1.42 (3.09)	< 0.001
40 to 50	1.69 (2.45)	1.7 (2.6)	1.69 (2.69)	0.519	2.6 (4.15)	0.98 (3.5)	1.36 (3.55)	< 0.001
50 to 60	1.64 (2.91)	1.62 (3.02)	1.6 (3.15)	0.48	2.5 (4.39)	0.67 (3.64)	1.41 (3.78)	< 0.001
60 to 70	1.53 (3.47)	1.54 (3.52)	1.51 (3.65)	0.482	2.5 (4.71)	0.58 (3.81)	1.47 (3.96)	< 0.001
70 to 80	1.42 (3.95)	1.43 (3.97)	1.38 (4.13)	0.519	2.66 (5.03)	0.7 (4.18)	1.48 (4.11)	< 0.001
80 to 90	1.26 (4.37)	1.23 (4.39)	1.19 (4.54)	0.55	2.72 (5.19)	0.94 (4.51)	1.64 (4.43)	< 0.001
90 to 100	1.01 (4.72)	0.95 (4.75)	0.95 (4.9)	0.579	2.81 (5.42)	1.25 (4.88)	1.92 (4.76)	< 0.001
100 to 110	0.72 (5.0)	0.64 (5.02)	0.61 (5.19)	0.633	3.14 (5.93)	1.46 (5.34)	2.13 (5.13)	< 0.001
110 to 120	0.29 (5.2)	0.33 (5.22)	0.29 (5.41)	0.755	3.43 (6.46)	1.44 (6.5)	2.21 (6.03)	< 0.001

* One-way analysis of variance.

CR, cruciate-retaining; MC, medial congruent; UC, ultra-congruent.

### Medial and lateral femoral roll back

**Fig. 4:**
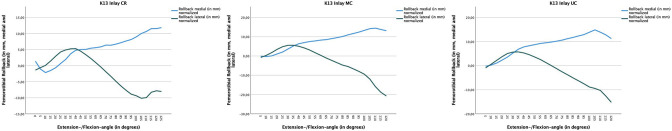
The normalized femoral roll back (medial compartment in blue, lateral in compartment in green) is shown using different inserts. The cruciate-retaining insert (left) shows a paradoxical roll forward in the first 30° of flexion and then initiates the actual backward movement in the lateral compartment. In contrast to the medial congruent (MC; middle) insert and ultra-congruent (UC; right) insert, there is a slight progression of the medial roll forward in deep flexion. The medial femoral condyle also remains relatively far anterior with the MC and UC insert. In this example, deep flexion in the MC and UC insert shows a regular, pronounced roll back on the lateral side. In a comparison of all 15 knee joints, however, this roll back is not detectable; here, the kinematics are more restrictive.

Across all 15 knee joints, medial and lateral femoral roll back showed significant differences between the inserts (p < 0.001, one-way analysis of variance and Brown-Forsythe test). The CR insert had a medial roll back of 19.53 mm (standard deviation (SD).7 5) on average and a lateral roll back of 13.62 mm (SD 8.36). The MC insert had a medial roll back of 20.51 mm (SD 8.11) and lateral roll back of 15.1 mm (SD 8), and the UC insert had a medial roll back of 19.81 mm (SD 7.96) and lateral roll back of 14.58 mm (SD 8.18). The MC insert was found to have the most restrictive kinematics with a span of 37.57 mm (medial) and 36.34 mm (lateral), the CR insert was found to have the least restrictive kinematics with a span of 42.21 mm medial and 37.88 mm lateral. The ranges also showed significant differences (CR vs MC; MC vs UC; CR vs UC). The values of knee number 13 as an example are summarized in Figure 4. A very pronounced paradoxical roll forward occurs on the medial side and thus there is only a “relative” roll back in the overall view, whereby the lateral compartment initiates an actual roll back between 60° and 65°. In view of all inserts, a recognizable, expectable pattern cannot be derived (e.g. typical MC insert behaviour with medially higher constraint and free lateral motion pattern).

### Femorotibial rotation

**Fig. 5:**
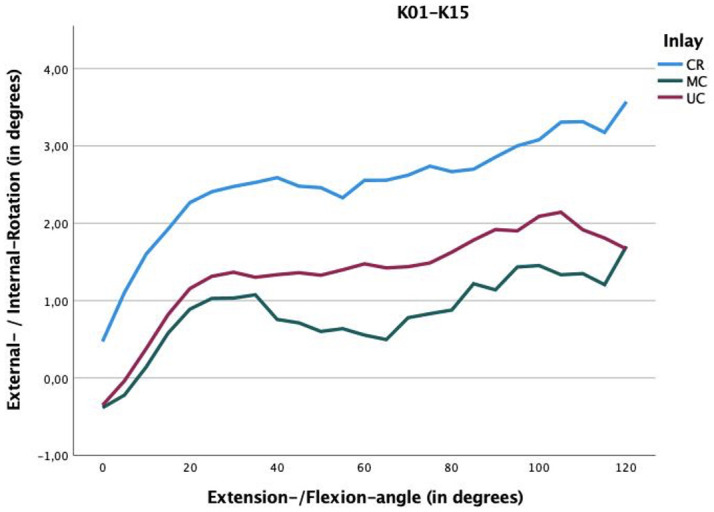
The rotation in the joint between femur and tibia was recorded. The medial congruent insert shows the highest constraint. Surprisingly, it was also higher than the ultra-congruent insert, which rotates on average 1.31° internally (femur versus tibia). The inserts also show significantly different values in all flexion groups (intervals of 10°) (p < 0.001, one-way analysis of variance).

Femorotibial rotation was found to be significantly different among these three inserts: the CR insert showed a mean femorotibial internal rotation of 2.45° (SD 4.75°) over the entire motion cycle, the MC insert had an internal rotation of 0.8° (SD 4.24°), and the UC insert an internal rotation of 1.31° (SD 4.15°). There were statistically significant differences among these inserts (CR vs MC, p < 0.001, one-way analysis of variance and Brown-Forsythe test; CR vs UC, p < 0.001; and MC vs UC, p < 0.001, one-way analysis of variance and Brown-Forsythe test). Table I shows the statistically significant differences in femorotibial rotation between the inserts at different degrees of flexion. The MC insert again shows the highest constraint of the three implants (Figure 5).

### Individual femoral, tibial, and patellar rotations

**Fig. 6:**
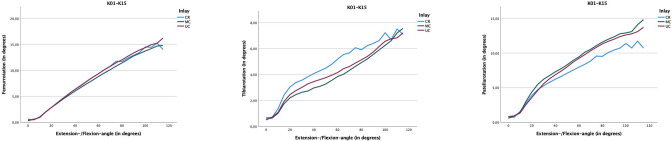
The individual rotations of the bones are shown (femur left, tibia middle, patella right), always referencing the respective initial value in 0° extension. Especially at the tibia, the highest constraint is shown with the medial congruent (MC) insert (compare also femorotibial rotation (Figure 5)). On the contrary, and to be understood as a compensation pattern, the highest rotation occurs at the patella with the MC insert. Here, the cruciate-retaining insert exhibits a significantly calmer pattern (although this in turn is associated with increased patella shifting (Figure 7 and Figure 8)).

Mean bone rotations for the femur, tibia, and patella are shown in Table II and in Figure 6. Like the femorotibial rotation (which occurred in the joint), the MC insert showed significantly higher constraint with the lowest femur and tibia rotation of the inserts. By contrast, the patella rotated the least with the CR insert and significantly more with the MC insert.

**Table II.** Mean rotation values of the bones, with rotations calculated in each case with an initial value in full extension.

Variable	CR	p-value*	Sig	MC	p-value*	Sig	UC	p-value*	Sig
Mean femur rotation, ° (SD)	8.02 (6.32)	< 0.001	†	7.68 (6.52)	< 0.001	‡	8.14 (6.59)	0.019	§
Mean tibia rotation, ° (SD)	4.54 (3.55)	< 0.001	†	3.7 (3.02)	< 0.001	‡	3.96 (3.1)	< 0.001	§
Mean patella, ° (SD)	6.92 (5.32)	< 0.001	†	8.19 (5.87)	< 0.001	‡	7.8 (5.76)	< 0.001	§

* If normal distribution of the data was found, the data were assessed using a one-way analysis of variance. The homogeneity of the variance was tested by Levene's test. If homogeneity could not be shown, the Brown-Forsythe test was used.

† Statistical significance for cruciate-retaining versus medial congruent.

‡ Statistical significance for medial congruent versus ultra-congruent.

§ Statistical significance for cruciate-retaining versus ultra-congruent.

CR, cruciate-retaining; MC, medial congruent; Sig, significance; UC, ultra-congruent.

**Fig. 7:**
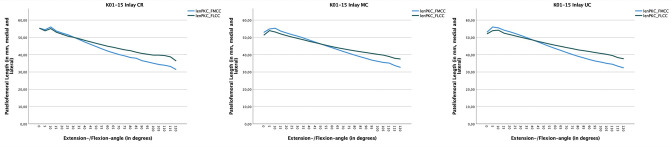
Patellar tracking is shown: in blue, the length between the patella centre and the centre of the medial femoral condyle (PKC-FMCC); analogously, in green, the length between the patellar centre (PKC) and the centre of the lateral femoral condyle (FLCC). If the length (medial or lateral) is proportionally shorter than on the opposite side, a shifting of the patella occurs. With the cruciate-retaining (CR) insert a medialization already occurs in early degrees of flexion (divergence between blue and green curve); in deep degrees of flexion there is therefore a greater shifting in total numbers. Again, the medial congruent insert shows a statistically significant higher constraint in comparison to the ultra-congruent and CR insert (Table III).

**Table III.** Patella lengths and differences between the underlying inserts.

Flexion, °	PKC-FMCC						PKC-FLCC					
	**CR**	p-value*	**MC**	p-value*	**UC**	**p-value†**	**CR**	p-value*	**MC**	p-value*	**UC**	p-value*
0	55.34	< 0.001	52.88	1.000	52.99	< 0.001	55.35	< 0.001	51.27	< 0.001	52.02	< 0.001
5	54.47	0.001	54.89	< 0.001	56.03	< 0.001	53.81	1.000	53.92	1.000	53.91	1.000
10	56.05	< 0.001	55.30	0.488	55.52	0.003	55.10	< 0.001	53.15	< 0.001	54.19	< 0.001
15	53.64	1.000	53.63	0.003	54.18	0.004	52.96	< 0.001	52.00	0.025	52.44	0.007
20	52.54	1.000	52.57	< 0.001	53.29	< 0.001	51.85	< 0.001	51.05	< 0.001	51.67	0.907
25	51.55	1.000	51.57	< 0.001	52.23	< 0.001	50.72	0.005	50.18	< 0.001	50.86	1.000
30	50.17	0.007	50.67	0.036	51.08	< 0.001	50.03	< 0.001	49.39	< 0.001	50.05	1.000
35	48.76	< 0.001	49.63	0.510	49.85	< 0.001	49.22	< 0.001	48.59	< 0.001	49.28	1.000
40	47.30	< 0.001	48.46	1.000	48.58	< 0.001	48.27	0.003	47.72	< 0.001	48.52	0.381
45	45.97	< 0.001	47.32	1.000	47.34	< 0.001	47.42	0.022	46.99	< 0.001	47.67	0.357
50	44.67	< 0.001	46.24	0.839	46.05	< 0.001	46.54	0.100	46.20	< 0.001	46.93	0.037
55	43.34	< 0.001	45.09	0.143	44.76	< 0.001	45.72	0.161	45.42	< 0.001	46.18	0.011
60	42.11	< 0.001	44.03	0.020	43.57	< 0.001	44.89	0.449	44.66	< 0.001	45.44	0.001
65	41.07	< 0.001	42.90	0.016	42.43	< 0.001	44.27	0.177	43.98	< 0.001	44.80	0.001
70	40.09	< 0.001	41.84	0.001	41.24	< 0.001	43.51	1.000	43.38	< 0.001	44.11	< 0.001
75	39.30	< 0.001	40.77	< 0.001	40.13	< 0.001	42.81	1.000	42.77	< 0.001	43.50	< 0.001
80	38.34	< 0.001	39.73	< 0.001	39.06	< 0.001	42.32	1.000	42.22	< 0.001	42.79	0.007
85	37.96	< 0.001	38.75	< 0.001	38.14	0.824	41.44	0.279	41.71	< 0.001	42.32	< 0.001
90	36.55	0.017	37.83	< 0.001	37.19	0.884	40.74	0.007	41.22	< 0.001	41.82	< 0.001
95	35.83	1.000	36.84	0.008	36.34	0.033	40.26	0.013	40.71	0.003	41.25	< 0.001
100	35.05	1.000	36.18	0.022	35.75	0.120	39.76	0.011	40.22	0.005	40.72	< 0.001
105	34.33	< 0.001	35.52	0.001	34.99	0.004	39.77	1.000	39.77	0.009	40.23	0.009
110	33.89	< 0.001	35.17	< 0.001	34.50	0.012	39.49	0.003	38.95	0.002	39.53	1.000
115	33.22	0.007	33.72	0.037	33.33	1.000	38.76	< 0.001	37.92	0.137	38.29	0.027
120	31.40	< 0.001	32.68	0.175	32.40	< 0.001	36.38	< 0.001	37.52	1.000	37.66	< 0.001

PKC-FMCC describes the length from the centre of the patella to the centre of the medial condyle.

PKC-FLCC describes the distance to the lateral condyle; if the distance to the medial decreases more than the lateral length, a medialization of the patella is assumed.

* One-way analysis of variance and Brown-Forsythe test.

† If normal distribution of the data was found, the data were assessed using a one-way analysis of variance. The homogeneity of the variance was tested by Levene's test. If homogeneity could not be shown, the Brown-Forsythe test was used.

CR, cruciate-retaining; MC, medial congruent; PKC-FLCC, patella knee centre (PKC) - femoral lateral condyle centre (FLCC); PKC-FMCC, patella knee centre (PKC) - femoral medial condyle centre (FMCC); UC, ultra-congruent.

**Fig. 8:**
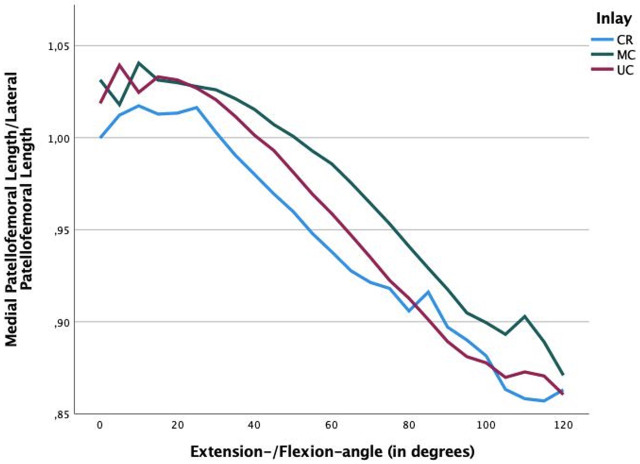
The quotient between medial and lateral distance of the patellar centre to the centre of the femoral condyle is shown. A quotient < 1 means that the patella shifts medially, while a quotient > 1 means that the patella shifts laterally (see Figure 6). The patella shows medialization earlier, at 30° flexion, when using a cruciate-retaining (CR) insert (quotient < 1), followed by the ultra-congruent (UC) insert at 40° flexion and the MC insert at 50° flexion. This trend also continues in deep flexion: thus, the CR insert shows a medialization quotient of 0.85 in 110° flexion, the UC insert a quotient of 0.87, and the medial congruent insert a quotient of 0.9.

On average, the patella rotates differently depending on the type of insert used: 6.92° (SD 5.32°) with a CR insert; 7.8° (SD 5.76°) with a UC insert; and 8.19° (SD 5.87°) with a MC insert. Notably, the curve of the CR insert flattens significantly from approximately 80° of flexion and does not exceed 12° of rotation, while the MC and UC inserts continue to rotate even in deep flexion (up to 15° rotation) (Figure 6).

The medial and lateral patellofemoral distances also statistically significantly differed (Table III). There is a significant patellar shift medially with the CR insert (Figure 7), and medialization (Figure 8) begins as early as between 30° and 35° of flexion. Medialization begins significantly later in the MC and UC inserts (UC at 40° of flexion, MC at 52° of flexion). In deep degrees of flexion (from 100°), the CR and UC inserts demonstrated significantly greater medialization than the MC insert (Figure 8).

## Discussion

From this study, we demonstrated that using a MC insert in CR TKAs offers significantly higher constraint than with an UC or CR insert. Thus, the kinematics with a MC insert exhibits the most restricted femorotibial rotation and individual rotation at the femur and tibia. Compensation appears to occur via the patella where we noted the highest patellar rotation occurring with the MC insert. However, the medialization of the patella using a MC insert was the lowest.

The kinematics with a CR insert demonstrated the highest overall laxity in the knee joint. The range of femoral roll back, femorotibial rotation, and the individual bony rotations were the highest with this implant. However, patellar rotation was found to be the lowest with significant medialization in flexion.

Expected femoral roll back could not be demonstrated with any insert. There was a pronounced paradoxical roll back of the used CR design. This was observed mainly medially, the lateral compartment was more stationary, and only at higher degrees of flexion was true lateral roll back identified.

### Limitations

Our study has limitations. First, it is a cadaveric study, thus in vivo data cannot be collected. Victor et al^15^ clarified the pronounced influence of the knee guiding musculature (especially the quadriceps muscle) on knee kinematics. Similar to all in vitro studies using cadaver specimens examined in a knee rig, the experiment only simulated a loaded squat. Nonetheless, passive movements also provide detailed information about the underlying kinematics. Second, the knee simulator is a passive testing machine, thus the effects of active muscle activities cannot be tested. This may be one reason for the femoral roll forward that was identified in our study. However, the kinematic fundamentals can be demonstrated with the above described comparisons. Nevertheless, the aim of future studies must be to generate preoperative and postoperative in vivo data to define the kinematic goals of implanting a TKA. Therefore, these results are only applicable to the mechanical alignment concept used in the study.

Notably, we identified paradoxical roll forward with each tibial insert, especially at the beginning of flexion. Consistently, lower values of paradoxical roll forward are reported in the literature,^7,8^ but this is due to the different measurement methods. In the usual in vivo testing, reference is always made to the deepest contact point between the insert and the femoral surface. In our study, the centres of the femoral condyles were defined, and this was related to the tibial plateau. Thus, in prior in vivo studies, the femoral geometry is considered uniform, or the different geometries are not taken into account (individually different J curve; difference between large and small knee joints). A standardized definition of femorotibial roll back should be established in the literature. The contact points may vary due to differences in femur geometry, but the centre of the medial or lateral femoral condyle seems to be more universally applicable.

The freedom that a CR insert gives, as evident from our figures, is certainly a contributory reason for the excellent long-term results. It is likely that low aseptic loosening rates are related to fewer forces being transferred to the implant-bone interface compared to other, more constraining prosthesis/insert types.^16^ Our study was able to show that the MC insert resulted in more restrictive kinematics of the lower limb. On the one hand, this may generate higher Forgotten Joint Scores, but on the other hand, higher forces are certainly transferred into the implant-bone or implant-cement interface.^17,18^ There, the question of an increased loosening rate is obvious. However, recent data by Christensson et al^19^ contradict this assumption: an increased migration rate when using a medial congruent insert could not be shown.

In addition to the femorotibial joint, some patellar parameters show interesting data. For example, the patella is more restricted in terms of tracking with the MC insert, but there is significantly higher patellar rotation compared with the CR insert. In contrast, there is almost no rotation with the CR insert, but there is increasing medialization at deeper degrees of flexion. A more detailed examination of the patella when using different inserts must be the subject of further studies. It also remains unclear whether these differences may be the cause of lowered outcome scores (anterior knee pain).

A final interesting point of the present study concerns the UC insert. The CS technique is becoming increasingly popular in the orthopaedic community,^20,21^ where the posterior cruciate ligament is resected during the procedure and an UC insert is used. As the name of the insert implies, high conformation and therefore higher constraint can be assumed. Surprisingly, in our study, the UC insert does not show the highest constraint in many variables whereas the MC insert does. In principle, the UC insert is close to the MC insert, but in femorotibial rotation and individual rotations the UC insert shows higher clearance than the MC insert. No explanation for this phenomenon is available in the literature. The uniform ball in socket configuration in the medial and lateral compartments with the UC insert may allow for greater mobility than the opposite design with the MC insert (medial ball in socket and lateral flat). Additionally, this is a single manufacturer’s tibial insert, it is possible that other implants may have different geometries and thus differing constraints associated with their implants.

It is noteworthy that the restrictions affect both the rotation between the tibia and femur (tibiofemoral rotation) and the rotation of each bone individually. Excessive rotation of both bones in the same direction (e.g. external rotation) with a smaller deviation from each other (tibiofemoral rotation) is also conceivable. However, the restrictions apply to both individual and global values.

In conclusion, in the present study, the higher constraint guidance of the MC insert compared with an UC or CR insert was demonstrated based on knee kinematics. Femoral roll back, femorotibial rotation, and single bony rotations were lowest with the MC insert. The patella showed less shifting with the MC insert, but there was significantly increased rotation. The presumed femoral roll back could not be shown in its pattern, but there was a pronounced paradoxical roll forward with a more stable lateral compartment and a more mobile medial compartment. Thus, a tighter guidance, or greater constraint with the MC insert can be assumed. However, it remains to be seen whether achieving native kinematics is associated with improved patient satisfaction and improved function.

**Take home message**

- Using a medial congruent (MC) insert in total knee arthroplasty restricts knee kinematics in femoral roll back, femorotibial rotation, and patella-shifting.

- The cruciate-retaining insert allows the greatest rotations (femorotibial and femorotibial roll back), but shows the least rotation at the patella.

- Surprisingly, the ultra-congruent insert also shows less constraint than the MC insert.

## Data Availability

The data that support the findings for this study are available to other researchers from the corresponding author upon reasonable request.
